# Childhood Cancer Mortality in India: Direct Estimates From a Nationally Representative Survey of Childhood Deaths

**DOI:** 10.1200/JGO.2015.000935

**Published:** 2016-04-13

**Authors:** Sumit Gupta, Shaun K. Morris, Wilson Suraweera, Lukasz Aleksandrowicz, Rajesh Dikshit, Prabhat Jha

**Affiliations:** **Sumit Gupta** and **Shaun K. Morris**, Hospital for Sick Children and University of Toronto; **Wilson Suraweera**, **Lukasz Aleksandrowicz**, and **Prabhat Jha**, St Michael’s Hospital, Toronto, Ontario, Canada; and **Rajesh Dikshit**, Tata Memorial Centre, Mumbai, India.

## Abstract

**Purpose:**

Although most children with cancer live in low- and middle-income countries, measurements of childhood cancer burden in such countries have been restricted to incidence rates from a few subnational cancer registries and mortality rates from vital statistics. We aimed to provide alternative burden estimates by using nationally representative longitudinal survey–derived mortality rates.

**Methods:**

We examined cancer deaths in childhood (1 month to 14 years of age) in the Million Death Study, a cohort of > 27,000 pediatric deaths in India on the basis of enhanced verbal autopsies. All deaths potentially due to childhood cancer were identified. Two pediatric specialists independently categorized deaths as definite, probable, possible, or unlikely cancer related. From definite and probable deaths, we estimated national and regional mortality rates attributable to childhood malignancies. Data on symptoms and health care–seeking behavior were abstracted from closed-ended questions and caregiver narratives.

**Results:**

Of 700 included deaths, 189 were classified as definite or possibly cancer related. The κ-statistic between reviewers was 0.75 (95% CI, 0.71 to 0.78). From these deaths, we estimated that in 2010, 13,700 were a result of childhood cancer in India, which led to a mortality rate of 37 (95% CI, 31 to 42) per million population per year, which exceeds many prior estimates of mortality and even some estimates of incidence. Disparities between mortality estimates were widest in northeast India and for brain tumors. A preponderance of male deaths was seen (male:female ratio, 1.6:1).

**Conclusion:**

The burden of childhood cancer in India is substantially higher than previously suggested. This information will aid advocacy for national strategies aimed at improving outcomes for Indian children with cancer.

## INTRODUCTION

Significant progress has been made in the treatment of pediatric cancer; > 80% of children in high-income countries (HICs) now achieve 5-year survival.^1,2^ These advances have not been fully realized in low- and middle-income countries (LMICs), where the majority of children with cancer reside.^3,4^ A major impediment to improving LMIC pediatric oncology outcomes is the paucity of data on the true burden of childhood cancer in these settings.^5^ This deficit hampers efforts to place childhood cancer on LMIC public health agendas and hinders the design and implementation of effective national childhood cancer strategies.^5^

In HICs, pediatric cancer incidence rates are determined by high-quality population-based cancer registries, whereas cancer mortality rates are usually determined through systems of death registration or vital statistics.^2^ Linkages between these two data sources provide a more complete picture of the cancer burden in a population. Estimations in LMICs is problematic given the paucity of registries and vital statistics. For example, in 2006, population-based cancer registries covered only 8% of populations in Asia and 11% in sub-Saharan Africa; when only high-quality registries are considered, proportions were 5% and 2%, respectively.^6^ Even where high-quality registries exist, poor access to health care and diagnos tic tools may still result in substantial underestimates of the actual incidence rates of childhood cancer. Where systems of death registrations exist, deaths still may be unreported and cause of death data lacking.^7^

We aimed to estimate the burden of childhood cancer in a specific LMIC (India) by using a methodology that focused on childhood cancer mortality and did not depend on cancer registries or vital statistics data bases. We used verbal autopsy reports from the Million Death Study (MDS), a unique, nationally representative, and longitudinal survey of > 14 million people.

## METHODS

### Million Death Study

Details of the MDS design, statistical and epidemiologic methods, and preliminary results pertaining to overall cancer mortality have been reported elsewhere.^8,9^ In brief, as part of the overall MDS, the Registrar General of India introduced a detailed verbal autopsy instrument into its nationally representative Sample Registration System (SRS) as a part of generating national vital statistics. The SRS comprised 6,671 small areas randomly chosen from approximately 1 million areas delineated by the 1991 census, each in turn comprising approximately 1,000 individuals. All individuals within the chosen areas were enumerated; subsequent births and deaths were documented every month by a local part-time enumerator and independently surveyed twice a year by one of 800 full-time Registrar General of India supervisors (nonmedical graduates). Deaths were documented between 2001 and 2003 and then between 2004 and 2014 in a new SRS sampling frame. Each surveyor who visited an SRS area recorded from appropriate informants a written narrative in the local language that described events preceding the death in addition to closed-ended questions related to key symptoms. Separate instruments were used for deaths in children age 1 month to 14 years on the basis of a World Health Organization multicountry validation study of verbal autopsy for common causes of childhood death.^10^

For each MDS death, each local language narrative and corresponding symptom data were electronically scanned and sent to two of 130 collaborating physicians trained in disease coding. These physicians independently assessed the most likely underlying cause of death by assigning a three-character code from the International Classification of Diseases, Tenth Revision (ICD-10).^11^ Disagreements were resolved by anonymous reconciliation; a third senior physician adjudicated persistent differences.

### Identification of Deaths as a Result of Childhood Cancer

We aimed to identify all pediatric (age 0 to 14 years) deaths due to malignancy reported in the MDS between 2001 and 2003. First, all MDS deaths that potentially resulted from childhood cancer were assembled by identifying those that met at least one of the following three criteria: final cause of death attributed to cancer, final cause of death attributed to a nonmalignant cause postreconciliation despite being attributed to cancer by one of the two initial assessing physicians, and nonmalignant causes of death a priori identified as difficult to differentiate from malignant causes by verbal autopsy. This latter criteria included the following causes of death: epilepsy (ICD-10 G40), neurologic deficits (ICD-10 R56), anemia (ICD-10 D50 to D64), sepsis or fever of unknown origin (ICD-10 A41, R50) if accompanied by pallor, and nutritional or ill defined (ICD-10 R96, R99) if accompanied by fever. Deaths within the first month of life were excluded given the rarity of cancer and high prevalence of infectious and congenital causes of mortality in this population.

Both the narratives of events that led to death and the closed-ended questions on symptoms, risk factors, disease history, and demographic characteristics were retrieved for all deaths that met these criteria. Professional translators translated the narratives into English. A pediatric oncologist (S.G.) and pediatric infectious disease specialist (S.K.M.) independently reviewed and classified each cause of death into one of four categories: definite cancer (proven by diagnostic testing), probable cancer (likely given clinical description and epidemiology but unproven by diagnostic testing), possible cancer (possible given clinical description and epidemiology but other nonmalignant causes also likely), and unlikely or definitely not cancer (other nonmalignant causes likely or confirmed by diagnostic testing). Reviewers were blinded to the original causes of death assigned in the MDS. Discrepancies were resolved through discussion. Agreement was assessed by using the κ statistic.^12^

Subsequent analyses were restricted to definite or probable deaths from cancer. Several data variables were abstracted from the narratives, which included symptom duration, type of treatment received, note of caregiver financial difficulties, and location of death. The highest level of health care accessed was also abstracted, with hospital-based care superseding care delivered by local health care workers. Hospitals were categorized as governmental, private, or other/unknown.

### Estimation of Total Number of Pediatric Cancer Deaths

The absolute number of deaths from cancer in childhood at a national level was derived by applying age- and sex-specific proportions of pediatric cancer deaths in the 2001 to 2003 MDS to 2010 United Nations estimates of all-cause pediatric deaths in India.^13-16^ These estimates were chosen due to proximity to the 2011 census. Despite census data that referred to population counts and not deaths, this estimate allowed us to correct for slight undercounts in total SRS-reported death rates. This correction process has been described previously.^17^ As reported previously, deaths missed by the MDS (approximately 12%) were due to family outmigration or from incomplete field records.^18^ Total pediatric deaths were portioned into region-specific total deaths by using the relative SRS pediatric deaths for 2007 to 2009.^19^ The age-sex–specific proportion of pediatric cancer deaths to total pediatric deaths in the current survey were weighted for the sampling probability of population selection for each rural or urban stratum per state, although such weighting made little difference because the study was nationally representative. Crude mortality rates per million population were calculated. Rates were standardized to the world population for comparison with other estimates.^20^

SRS enrollment was voluntary, and its confidentiality and consent procedures are defined as part of the Registration of Births and Deaths Act, 1969. Oral consent was obtained in the first SRS sample frame. Families were free to withdraw from the study; compliance was nonetheless > 95%. The study was approved by the review boards of the Post-Graduate Institute of Medical Education and Research, St Michael’s Hospital, and the Indian Council of Medical Research. Statistical analyses were performed with SAS version 9.4 software (SAS Institute, Cary, NC).

## RESULTS

**Fig 1:**
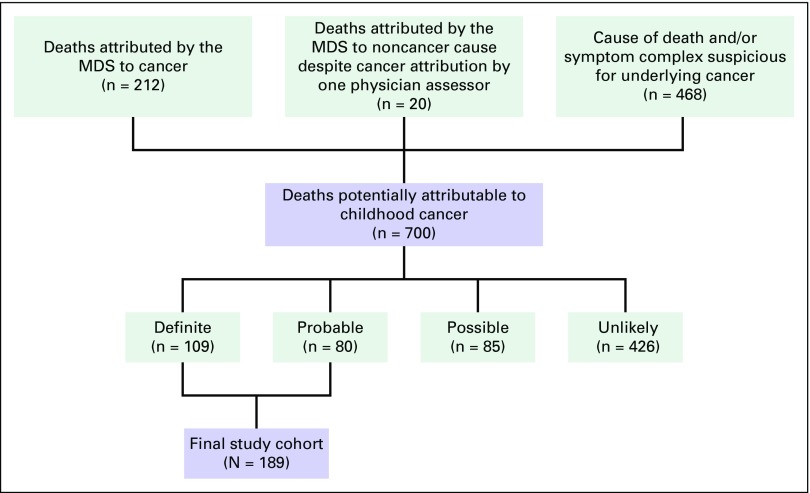
Identification of Million Death Study (MDS) deaths attributable to childhood cancer.

A total of 700 deaths met initial MDS inclusion criteria: 212 attributed to cancer, 20 to a final nonmalignant cause by reconciliation or adjudication despite one reviewing physician’s attribution to cancer, and 468 to causes of death or symptoms similar to a potential malignancy. After categorization and resolution of discrepancies, 189 deaths were classified as either definitely (n = 109) or probably (n = 80) a result of cancer. These 189 deaths comprised the final study cohort. Eighty-five deaths were categorized as possibly cancer related, with the remaining 426 classified as unlikely or definitely not cancer related. The weighted κ between the two reviewers’ categorizations was 0.75 (95% CI, 0.71 to 0.78). The flow of case selection is illustrated in Figure 1. Forty-nine deaths originally attributed to cancer by the MDS were categorized as either possibly or unlikely/definitely not cancer; 26 deaths originally attributed as nonmalignant causes by the MDS were categorized as definitely or probably cancer related. The most common causes were fever of other and unknown origin (n = 6 [23%]), ill defined and unspecified (n = 5 [19%]), and other anemias (n = 5 [19%]). The full list of MDS-attributed causes of death for these 26 patients are shown in the Data Supplement. The demographic characteristics of the final study cohort are listed in Table 1.

**Table 1:**
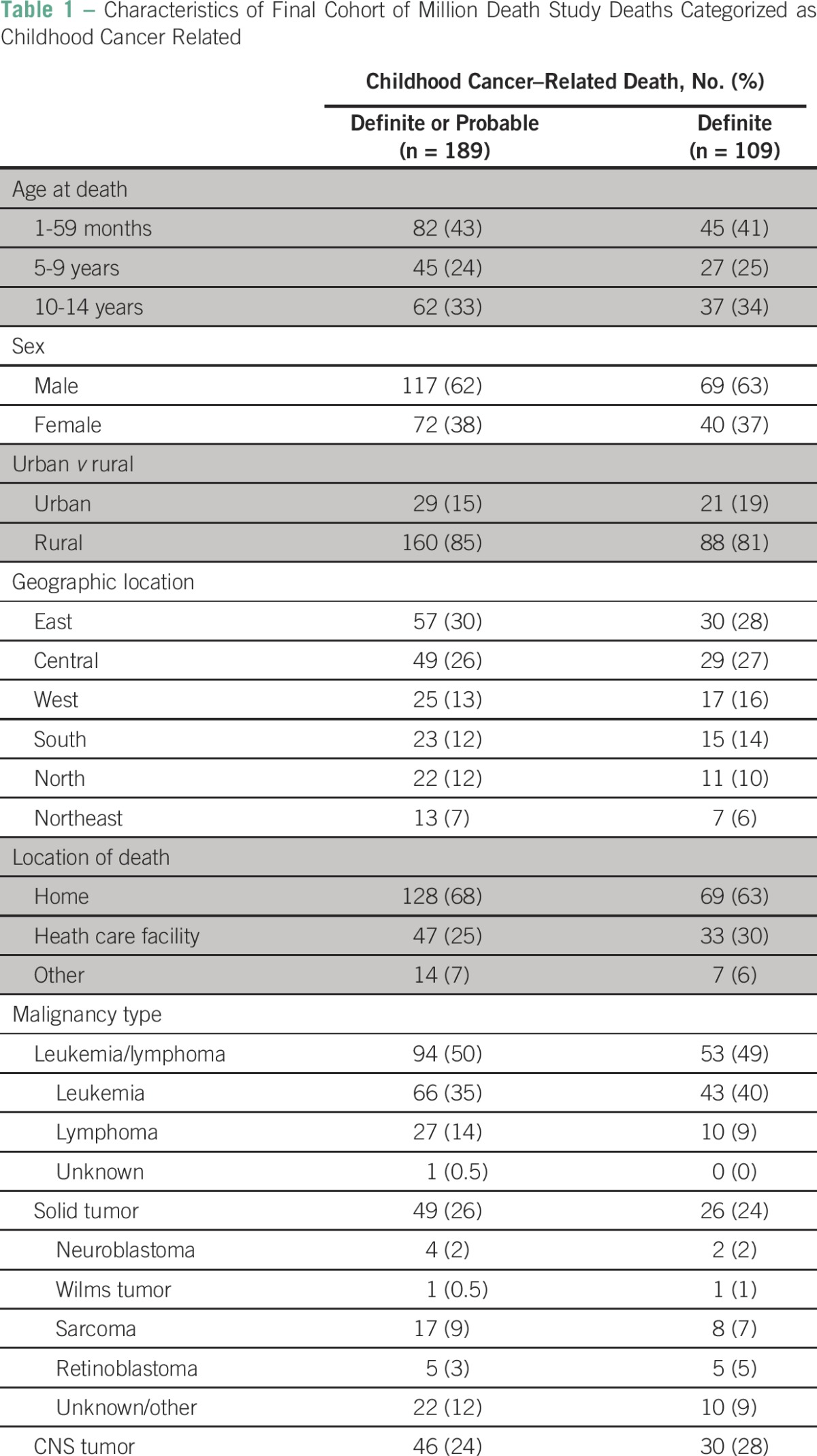
Characteristics of Final Cohort of Million Death Study Deaths Categorized as Childhood Cancer Related

**Fig 2:**
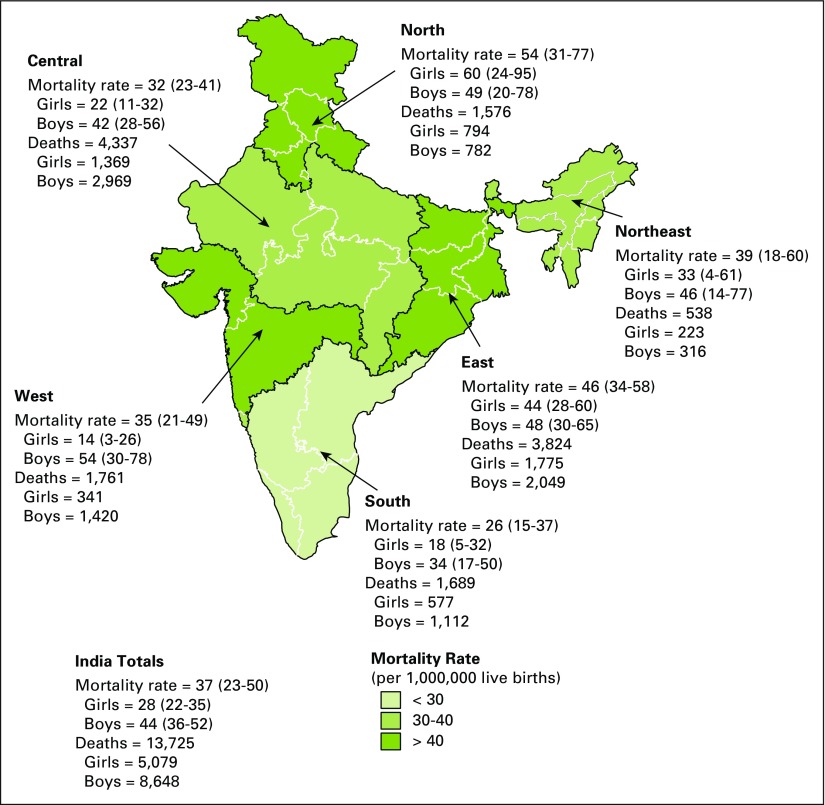
Estimated national-level deaths and mortality rates due to childhood cancer in India by region.

A total of 13,700 (95% CI, 11,800 to 15,700) pediatric cancer deaths were estimated in India in 2010. The national rate of pediatric cancer deaths was 37 (95% CI, 31 to 42) per million population. When standardized according to the world standard population for children age 0 to 14 years, a mortality rate of 39 (95% CI, 33 to 44) per million population was obtained. Regional deaths and cancer mortality rates are illustrated in Figure 2. The number of study deaths and corresponding national-level death estimates and mortality rates by cancer subgroups are shown in Table 2. Comparisons to selected Indian and HIC population-based cancer registries are shown in Table 3.

**Table 2:**
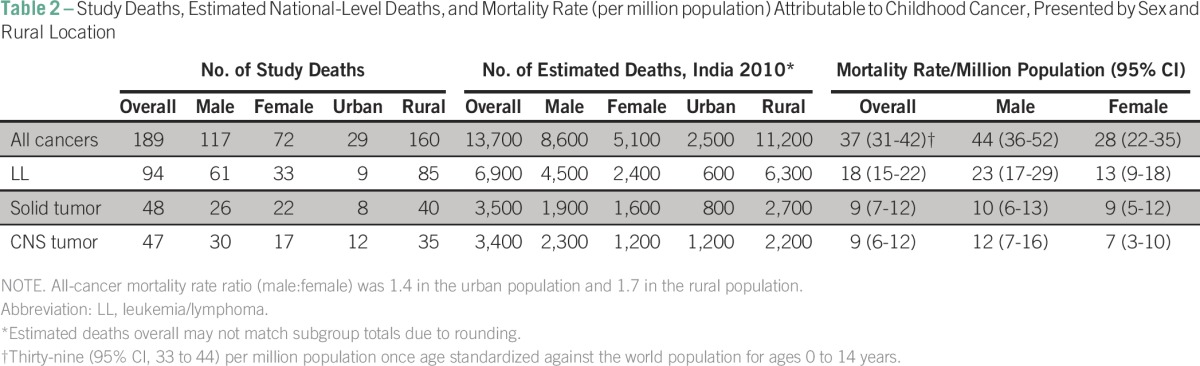
Study Deaths, Estimated National-Level Deaths, and Mortality Rate (per million population) Attributable to Childhood Cancer, Presented by Sex and Rural Location

**Table 3:**
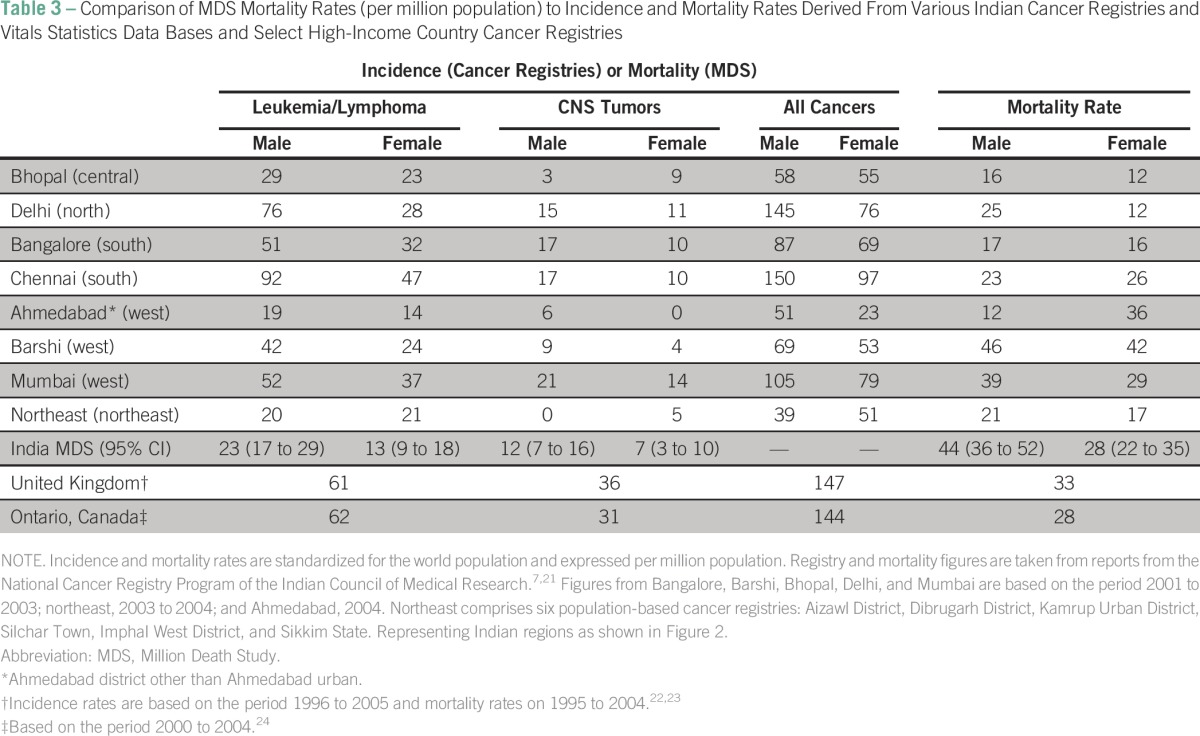
Comparison of MDS Mortality Rates (per million population) to Incidence and Mortality Rates Derived From Various Indian Cancer Registries and Vitals Statistics Data Bases and Select High-Income Country Cancer Registries

A total of 158 (83.4%) MDS patients experienced symptoms preceding death for > 1 month’s duration. Some level of health care was received by 183 (96.8%) patients before death (Table 4). Treatment of any form was received by 153 (81.0%) patients. The delivery of blood products was noted in 28 (14.8%) patients and antibiotics in 19 (10.1%). Cancer-directed therapy was rarely delivered: Only 36 (19.0%) narratives noted the receipt of surgery, eight (4.2%) the receipt of chemotherapy, and three (1.6%) the receipt of radiation. One hundred eleven (58.7%) narratives noted the delivery of some medical therapy without further detail. Traditional or alternative therapy was noted in 16 (8.5%) patients and financial difficulties in 19 (10.1%). Death occurred at home in the majority of narratives (n = 109 [57.7%]). A further 49 (25.9%) deaths occurred in health care facilities, although one (0.5%) occurred en route to the hospital. The location of death was unknown in the remaining 30 (15.9%) narratives. No significant differences were found by sex in symptom duration (> 1 month: boys, 95 of 116 [81.8%]; girls, 63 of 72 [(87.5%]; *P* = .31), receipt of any treatment (boys, 96 of 117 [82.1%]; girls, 57 of 72 [79.2%]; *P* = .62], or location of death (deaths at home: boys, 68 of 97 [70.1%]; girls, 41 of 61 [67.2%]; *P* = .70).

**Table 4:**
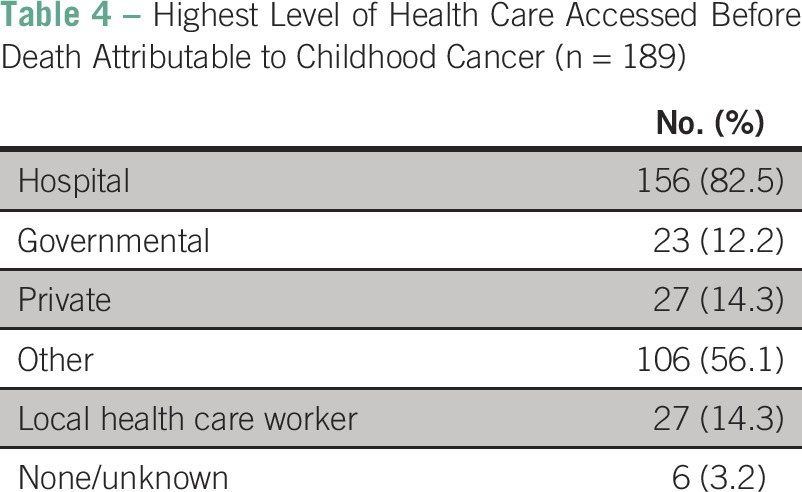
Highest Level of Health Care Accessed Before Death Attributable to Childhood Cancer (n = 189)

## DISCUSSION

By using verbal autopsies from a nationally representative cohort and a rigorous process of reassessment of the causes of death, we aimed to characterize the burden of childhood cancer mortality in India. We found higher mortality rates than reported in the past and, in some instances, that even exceeded estimates of incidence provided by population-based cancer registries. Although this may reflect true differences between the national mortality rate and rates in specific smaller geographic areas with nonrepresentative populations, underdiagnosis and under-registration are also likely to play a role.

We determined an overall age-standardized mortality rate due to pediatric cancer of 39 (95% CI, 33 to 44) per million population per year. This rate is higher than those reported from most jurisdictions (Table 3). This disparity was most pronounced in certain malignancies and in certain regions. For CNS tumors, the MDS-derived mortality rate was nine per million population per year, a rate distinctly higher than even the incidence rates reported by several cancer registries. Explanatory factors include the difficulty in diagnosing CNS tumors without expensive diagnostic modalities, such as computed tomography scans, and the variation between registries in their inclusion of benign CNS malignancies. Combining information from six local administrative areas, the Indian Council of Medical Research reported a mortality rate attributable to childhood cancer in northeast India of 19 per million population per year.^21^ The equivalent MDS-derived rate for the northeast region is more than twice as high at 39 (95% CI, 18 to 60) per million population.

Even where high-quality cancer registries exist, multiple steps are required to capture pediatric cancer diagnoses.^25^ Caregivers must seek medical attention, and health care workers must refer patients to tertiary centers capable of diagnosing malignancies. Only upon a correct cancer diagnosis can a tertiary center register the patient case. Vital statistics depend on similar steps. Breaks in this chain of events may occur at any step, which leads to underestimates of true incidence or mortality rates.^25^ Such underestimates are likely to be substantial in settings with barriers to accessing high-quality health care. These issues have been noted not only in India but also across various LMICs.^26,27^

Our methodology that used MDS data overcomes many, but not all of these limitations. First, given the nationally representative sampling of the MDS, national estimates of childhood cancer burden are possible. Second, all causes of death, in addition to cancer, are captured. Consequently, the MDS-based analyses will have captured several groups of children with cancer missed by cancer registries: those with confirmed malignancies diagnosed at facilities that do not report to registries and those whose signs and symptoms were a result of a malignancy but who did not undergo diagnostic testing. Efforts to capture this latter group included both the initial review of verbal autopsies by MDS physicians and the independent re-review of candidate deaths by two pediatric specialists. This resulted in the reclassification of a small, but significant number of deaths.

Despite this rigorous re-review, misclassification of deaths is still certain to have played a role in the current analyses. Such misclassification is of particular concern in hematologic malignancies, in which the presenting symptoms (fever, malaise, hemorrhage) have substantial overlap with those of more common, infectious causes of death. Other authors have noted that disparities in registry-derived incidence rates of pediatric leukemia between LMICs and HICs are wider than when comparing the incidence of more recognizable tumors that present with, for example, enlarging masses.^25^ Despite these limitations, these figures are, to our knowledge, the most nationally representative derived from primary data in India to date. With an appropriate degree of uncertainty because of the possibility of misclassification, these figures suggest a burden of childhood cancer mortality in India that is substantially higher than previously reported.

Several additional findings merit discussion. First, the male to female ratio among deaths attributable to childhood cancer was 1.6:1. By contrast, previous MDS analyses of childhood mortality have identified a marked excess of female deaths.^14^ For example, death as a result of both diarrheal diseases and pneumonia was nearly 50% higher in girls than in boys, whereas measles deaths were nearly 70% greater in girls than in boys.^15,16^ This preponderance of female deaths has been attributed to the impact of sex preferences on health care–seeking behavior; previous studies have shown a correlation between areas with higher excess mortality rates among young girls and those with lower female:male sex ratios for second births after a boy, a marker of the presence of sex-selective abortion.^14,28^ The same phenomenon is likely responsible for the male preponderance seen in the current study. Preferentially accessing health care for boys may lead to lower mortality rates for conditions where treatment is both relatively simple to deliver and effective (eg, diarrheal diseases). In settings where childhood cancer treatment is limited or unavailable, the same sex-based health care–seeking preference for cancer may lead to an increased probability of a correct cancer diagnosis being made without increasing the probability of cure.

Second, the MDS verbal autopsy narratives indicated that the duration of symptoms experienced by cohort patients exceeded 1 month in > 80% of deaths. This finding supports previous conjecture that delayed diagnosis is a significant issue in LMIC children with cancer.^29^ Although the impact of prolonged times to diagnosis in HICs is still under debate, previous authors have suggested that they represent a major barrier to cure in LMICs, which results in advanced stage and poor performance status at diagnosis.^29-31^ Similarly, although nearly all of the MDS cohort (96.8%) accessed some level of health care, only a small number received any cancer-directed therapy. Though these numbers should be considered with caution given their dependence on open-ended caregiver narratives and the inability to generalize them to children who experienced cure (see next), they nonetheless provide a first population-based look at the trajectory experienced by LMIC children who succumb to cancer.

Third, although the ability of verbal autopsies and multiple independent physician coders to identify major causes of death correctly has been established in both adults and children,^10,32^ the identification of uncommon causes of mortality may be more difficult. Our technique, which involved central re-review of deaths by the appropriate specialist physicians, represents a method by which rare causes of death may be more accurately captured and thus extends the utility of verbal autopsies. Although the external validity of our methodology is impossible to determine given the absence of a gold standard, the high rate of agreement between the two reviewers (κ = 0.75; 95% CI, 0.71 to 0.78) provides reassurance.^33^ Of note, the two reviewers represented different areas of specialty: pediatric cancer (the condition of interest) and pediatric infectious disease (the most likely area of misclassification). Concordance between the two specialists, therefore, lends greater face validity. Similar techniques were used to identify deaths attributable to rabies and may be used in future studies of rare diseases.^34^

Additional limitations should be noted. The absolute number of identified deaths was relatively small, particularly when subdivided by geographic region. Thus, although geographic variation in childhood cancer mortality rates are plausible given large socioeconomic differences across India, they should be viewed as hypothesis generating only and await confirmation in future studies. Similarly, disparities by cancer type should be viewed with a degree of caution. The current cohort did not include children who survived cancer; therefore, we were unable to estimate childhood cancer incidence rates in India. Finally, limitations of verbal autopsies include a potential inability to capture homeless populations, dependency on caregiver recall accuracy, and appropriate translation.

In conclusion, these analyses provide national estimates of the burden of childhood cancer mortality in India that are substantially higher than those previously documented. These rates can be used by childhood cancer advocates and policymakers to both encourage and design national childhood cancer strategies and to improve the outcomes of LMIC children with malignancies.
